# Effect of High-Fat and Low-Fat Dairy Products on Cardiometabolic Risk Factors and Immune Function in a Low Birthweight Swine Model of Diet-Induced Insulin Resistance

**DOI:** 10.3389/fnut.2022.923120

**Published:** 2022-06-17

**Authors:** Yongbo She, Kun Wang, Alexander Makarowski, Rabban Mangat, Sue Tsai, Benjamin P. Willing, Spencer D. Proctor, Caroline Richard

**Affiliations:** ^1^Division of Human Nutrition, Department of Agricultural, Food and Nutritional Science, University of Alberta, Edmonton, AB, Canada; ^2^Metabolic and Cardiovascular Diseases Laboratory, Department of Agricultural, Food and Nutritional Science, University of Alberta, Edmonton, AB, Canada; ^3^Department of Medical Microbiology and Immunology, University of Alberta, Edmonton, AB, Canada

**Keywords:** dairy fat, cardiovascular disease (CVD), obesity, insulin resistance, immune function, swine model

## Abstract

Although dairy intake has been shown to have a neutral or some beneficial effect on major cardiometabolic risk factors, the impact of dairy, and especially dairy fat, on immune function remains to be investigated. To understand the effect of consuming dairy fat on cardiometabolic risk factors and immune function, we used an established low birthweight (LBW) swine model of diet-induced insulin resistance to compare high-fat and low-fat dairy products to a control high-fat diet (CHF). LBW piglets were randomized to consume one of the 3 experimental HF diets: (1) CHF, (2) CHF diet supplemented with 3 servings/day of high-fat dairy (HFDairy) and (3) CHF diet supplemented with 3 servings/day of low-fat dairy (LFDairy). As comparison groups, normal birthweight (NBW) piglets were fed a CHF (NBW-CHF) or standard pig grower diet (NBW-Chow). A total of 35 pigs completed the study and were fed for a total of 7 weeks, including 1 week of CHF transition diet. At 12 weeks of age, piglets were euthanized. Fasting blood and tissue samples were collected. *Ex vivo* cytokine production by peripheral blood mononuclear cells (PBMCs) stimulated with pokeweed (PWM), phytohemagglutinin (PHA) and phorbol myristate acetate-ionomycin (PMA-I) were assessed. As expected, LBW-CHF piglets showed early signs of insulin resistance (HOMA-IR, *P* model = 0.08). Feeding high-fat dairy products improved fasting plasma glucose concentrations more than low-fat dairy compared to LBW-CHF (*P* < 0.05). Irrespective of fat content, dairy consumption had neutral effect on fasting lipid profile. We have also observed lower production of IL-2 after PWM and PHA stimulation as well as lower production of TNF-α and IFN-γ after PWM stimulation in LBW-CHF than in NBW-Chow (all, *P* < 0.05), suggesting impaired T cell and antigen presenting cell function. While feeding high-fat dairy had minimal effect on immune function, feeding low-fat dairy significantly improved the production of IL-2, TNF-α and IFN-γ after PWM stimulation, IL-2 and IFN-γ after PHA stimulation as well as TNF-α after PMA-I stimulation compared to LBW-CHF (all, *P* < 0.05). These data provide novel insights into the role of dairy consumption in counteracting some obesity-related cardiometabolic and immune perturbations.

## Introduction

The immune system consists of two major arms, namely innate (e.g., skin, mucosal, myeloid immune cells) and adaptive (T and B lymphocytes) immunity, that together mount a regulated protective immune response to foreign stimuli. A proper immune response to infections is influenced by genetics, age, sex, smoking, alcohol, as well as the presence of obesity and nutrition (1, 2). In the context of obesity, alterations in the secretory output from adipose tissue is characterized by an increased release of cytokines and chemokines resulting in an excessive recruitment, infiltration and polarization of macrophages toward a pro-inflammatory phenotype (i.e., M1-like macrophages) (3). On the other hand, obesity-related metabolic complications are also associated with an increased intestinal permeability that further promotes systemic inflammation through the translocation of endotoxin into circulation (4, 5). Obesity-induced inflammation is also known to impair insulin signaling which contributes to insulin resistance and dyslipidemia and ultimately to the progression of cardiovascular disease (CVD) (6). We have previously demonstrated that individuals with obesity and type 2 diabetes (T2D) have an impaired T cell and neutrophil response following mitogen stimulation (7) compared to metabolically healthy individuals with obesity. This suggests that regardless of the obesity status, hyperglycemia and/or insulin resistance further impairs immune function. More recently, obesity and T2D have also been identified as major risk factors for severe COVID-19 outcomes (8) contributing to the body of evidence demonstrating that obesity impairs immune function.

Nutrients play a critical role in modulating obesity-related cardiometabolic perturbations and immune dysfunction. Fatty acids are key components in modulating blood lipid profile, while also exerting pro/anti-inflammatory activities (9, 10). In this context, saturated fatty acids (SFA) are generally considered pro-inflammatory, yet not all SFA exert the same pro-inflammatory properties (9). Medium-chain SFA may be beneficial for weight loss and major CVD risk factors (11, 12). Pentadecanoic acid (C15:0), the ruminant-derived odd-chain SFA, has been shown to exert anti-inflammatory activity (13) and be inversely associated with variables of insulin resistance and T2D (14). Additionally, the natural *trans*-fat vaccenic acid (18:1 *trans*-11) has been found to be beneficial to immune function (15). Recent work from our group has also demonstrated the promising role of dietary choline, particularly in the forms of phosphatidylcholine (PC) and sphingomyelin (SM), at improving the immune system development and function in rats (16, 17). Dairy products are unique and possess a complex food matrix that contains significant amounts of choline and medium-chain SFA along with other important nutrients. Still, many consumers are avoiding dairy products due to the perception that diets high in SFA may lead to adverse effects on health. In contrast, emerging evidence now point toward a neutral to potentially beneficial effect of total dairy and dairy fat intake on most CVD risk factors including blood pressure, inflammation, T2D and dyslipidemia (18–22).

The debate continues on the contribution of dairy fat *per se* on overall cardiometabolic health. One of the major reasons for this is that most studies to date have not considered the complex food matrix when comparing high-fat vs. low-fat dairy products (i.e., comparing milk to cheese or milk to butter) (23, 24). Few studies have assessed the effect of dairy foods and dairy fat, considering the importance of the food matrix on immune function. Given the glucose, insulin and immune modulatory effects attributed to certain nutrients found in dairy foods and dairy fat, the overall aim of this study was to determine the effect of consuming 3 servings/day of high-fat vs. low-fat products on obesity-related cardiometabolic perturbations and immune function in a pre-established swine model of insulin resistance. Commercially available high-fat and low-fat dairy products of similar food matrix were chosen. We hypothesized that consumption of 3 servings/day of dairy products, irrespective of the fat content, would exert a neutral effect on the lipid profile. We also proposed that feeding high-fat dairy may provide greater benefits on glucose metabolism, inflammation and immune function due to the presence of unique bioactive nutrients in dairy fat.

## Materials and Methods

### Animals and Housing

All piglets were born and raised at the Swine Research Technology Center (SRTC), Department of Agricultural, Food and Nutritional Science, University of Alberta, Canada. Piglets used in the present study are offspring of a cross of Duroc boar and Large White-Landrace sow. Only male piglets were used in this study, as they were more susceptible to develop early insulin resistance. The selection criteria were previously described (25). Briefly, a mean litter weight and standard deviation was determined to find a 95% confidence interval (CI), categorizing piglets as low birthweight (LBW, less than the 95% CI) or normal birthweight (NBW, within or above the 95% CI). Water, temperature control and routine health checks were performed daily by trained staff at the SRTC. Piglets were socialized regularly by trained staff to minimize stress response during subsequent experimental procedures. All study protocols were approved by University of Alberta Animal Care and Use Committee and in accordance with regulations of Canadian Council of Animal Care (Protocol AUP00001184).

### Study Design

**Figure 1:**
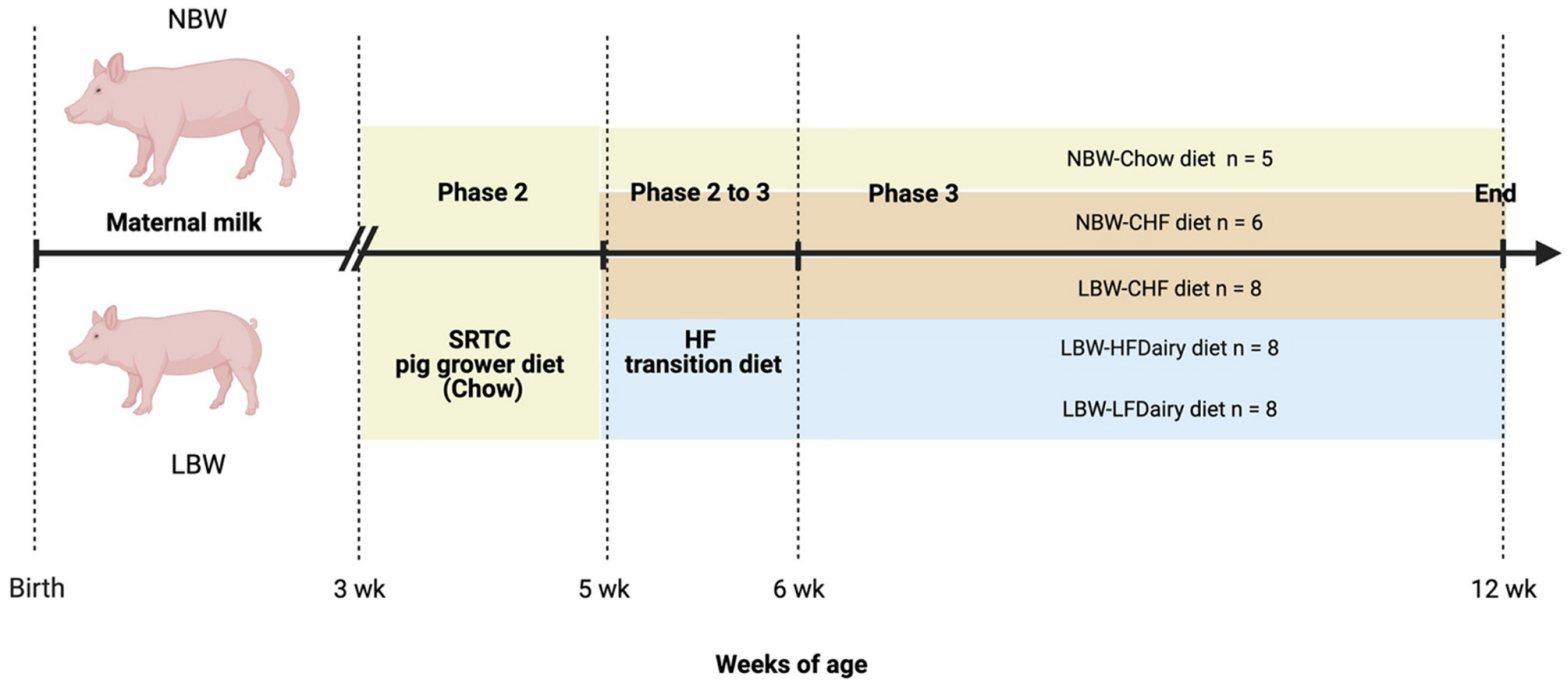
Study design to investigate the effects of consuming dairy fats on cardiometabolic risks and immune function in swine model of IR (figure created with BioRender). CHF, control high-fat diet; HF, high-fat; HFDairy, high-fat dairy diet; LBW, low birthweight; LFDairy, low-fat dairy diet; NBW, normal birthweight; SRTC, swine research technology center.

All piglets were weaned at 3 weeks of age with a SRTC pig grower diet (Chow) until 5 weeks of age. At 5 weeks of age, LBW piglets were randomized to consume one of the 3 experimental diets: (i) Control High-Fat (CHF), (ii) CHF supplemented with 3 servings per day of high-fat dairy products (HFDairy) or (iii) CHF supplemented with 3 servings per day of low-fat dairy products (LFDairy). As comparison groups, NBW piglets were fed a Chow (NBW-Chow) or CHF diet (NBW-CHF). A total of 35 pigs (LBW-CHF *n* = 8, LBW-HFDairy *n* = 8, LBW-LFDairy *n* = 8, NBW-CHF *n* = 6, NBW-Chow *n* = 5) completed the study and were fed for a total of 7 weeks, including 1 week of CHF transition diet (Figure 1). At 12 weeks of age, pigs were fasted overnight, and fasting blood were collected before termination at the SRTC. Liver tissues were collected after.

### Diet

The detailed nutrient composition of the experimental Chow and CHF diet has been previously described (25, 26). Briefly, the respective percentages of energy (Kcal) derived from fats, carbohydrates and protein were 46% (mainly lard), 33% (with 17% calories from fructose specifically) and 21%. 1% w/w cholesterol was also added. In contrast, the Chow diet consisted of 14% total energy from fat, 69% total energy from carbohydrate and 17% total energy from protein. To understand the effect of dairy products and more specifically dairy fat, the CHF diet was supplemented with 3 servings for every 2,000 Kcal of either low- or high-fat dairy products. In the LFDairy diet, one serving of skimmed milk powder (0% fat; No Name^®^, Canada), plain yogurt (1% fat; Foremost Farms, Canada) and mozzarella cheese (18% fat; No Name^®^, Canada) was fed to each pig daily contributing to ~16% of total energy intake in that group (Table 1). In the HFDairy diet, one serving of whole milk powder (3.25% fat; Bulk Barn, Canada), plain yogurt (10% fat; Liberté, Canada) and mozzarella cheese (28% fat; No Name^®^, Canada) was fed to each pig daily contributing to ~28% of total energy intake in that group. The serving size for each dairy product was based on the previous Canada's food guide (version 2007) which consisted of 250 ml of milk or powdered milk (24 g low-fat milk powder, 33.5 g high-fat milk powder), 175 ml of yogurt and 50 g of cheese regardless of their fat content. The number of servings provided per day was adjusted weekly proportionally to their increasing energy intake to maintain a constant ratio of 3 servings per 2000 Kcal intake. All products were available and purchased *via* retail outlets fresh every 10 days. Since pigs in both dairy groups did not lower their consumption of the experimental CHF diet, these two groups were pair-fed to the group receiving the experimental CHF diet only. Therefore, less experimental CHF diet was added to the feeder to compensate for the amount of calories coming from dairy products so that energy intake would be consistent across all three LBW groups. Body weight and food intake were measured and adjusted weekly.

**Table 1:** Nutrient composition of dairy products per 2,000 Kcal diet.

	**Amount per serving (g)**	**Energy (kcal)**	**Protein (g)**	**Fat (g)**	**Carbohydrate (g)**
Milk Powder, 3.25% MF	33	174.9	8.91	8.91	13.2
Greek Yogurt, 10% MF	175	200	6	17.5	5
Mozzarella Cheese, 28% MF	50	183.3	11.7	13.3	1.7
Milk Powder, 0% MF	24	90	9	0.18	12.6
Greek Yogurt, 1% MF	175	100	8	1.5	14
Mozzarella Cheese, 18% MF	50	150	15	8.3	1.7

*Nutrient composition was calculated based on serving size of Canada's Food Guide (2007) in a 2,000 Kcal diet. All dairy products were purchased from local distributor. MF, milk fat; Greek yogurt (1% MF), Foremost Farms, Canada; Greek yogurt (10% MF), Liberté, Canada; Milk powder (0% MF), No Name^®^, Canada; Milk powder (3.25% MF), Bulk Barn, Canada; Mozzarella cheese (18% MF); No Name^®^, Canada; Mozzarella cheese (28% MF), No Name^®^, Canada*.

### Tissues, Blood Processing, and PBMC Isolation

Fasting blood samples were collected in tubes containing EDTA, dipeptidyl peptidase 4 inhibitor (EMD Millipore, MA), and Complete^®^ general protease inhibitor (Sigma-Aldrich, USA) before being centrifuged at 3,000 rpm for 10 min to obtain plasma. Plasma was aliquoted and stored at −80°C until further analysis. To isolate PBMCs (peripheral blood mononuclear cells), 1% bovine serum albumin in phosphate-buffered saline was added to dilute and resuspend the buffy coat. Cell suspension was then layered over 5 ml histopaque and followed by centrifugation at 1,800 rpm for 30 min. PBMCs were recovered from the gradient interface, washed with 1% bovine serum albumin in phosphate-buffered saline, and reconstituted with 5% complete culture medium (RPMI 1640 media; Life Technologies, Burlington, ON, Canada). Prior to *ex vivo* analyses, a hemocytometer was used to count cells using trypan blue dye exclusion. All cell suspensions were then diluted to 1.25 ×10^6^ cells/ml. Additional PBMCs were frozen in 10% complete culture media (RPMI 1640 media; Life Technologies, Burlington, ON, Canada) with 10% DMSO at −80°C before transferred to liquid nitrogen. Samples of liver were collected and treated with ice-cold saline solution and snap frozen in liquid nitrogen prior to be stored at −80°C until further processing.

### Plasma Biochemical and Liver Fatty Acid Analysis

Fasting plasma concentrations of triglyceride (TG), total cholesterol (TC), low-density lipoprotein cholesterol (LDL-C), high-density lipoprotein cholesterol (HDL-C) and glucose were assessed using commercial enzymatic colorimetric kits (Wako Pure Chemicals, Tokyo, Japan) and as described previously (25, 26). Plasma insulin concentration was measured using commercial porcine-specific ELISA kits as per manufacturer's instructions (Mercodia, USA). Additional aliquots of plasma within the same group were mixed and measured for lipoprotein associated plasma TG and cholesterol fractions by fast protein liquid chromatography (FPLC) at the University of Alberta lipidomic core facility as described elsewhere (27). Fasting plasma inflammatory marker concentrations were assessed by porcine cytokine/chemokine 13-Plex Discovery Assay^®^ Array at Eve Technologies (Calgary, Canada).

Liver tissue fatty acid profile was assessed by gas chromatography equipped with a flame ionization detector (Agilent 8890). Briefly, 100 mg of liver tissue in 1 ml phosphate-buffered saline were treated with tissue homogenizer, and the homogenates were collected for analysis. Two hundred microliter homogenate was pipetted into glass tube and followed by conventional Folch extraction for total lipids. After overnight incubation at 4°C, the bottom layer of the lipid phase was dried under nitrogen gas. Methanolic KOH (1.5 ml) was then added followed by saponification at 110°C for 1 h. Methylation of fatty acids was conducted by adding 1.5 ml BF3 (boron trifluoride) and 1.5 ml hexane followed by incubation at 110°C for 1 h. After cooling, 1 mL of deionized water was added. The top layer was then collected, dried, reconstituted with hexane and then stored at −80°C until further analysis. All fatty acids in liver were expressed as percentage of total identified fatty acids.

### PBMC Membrane Phospholipid Analysis

Phospholipids in PBMC membranes were analyzed by high performance liquid chromatography (HPLC) at University of Alberta Lipidomic Core Facility. Briefly, isolated cells were resuspended in 200 μl phosphate-buffered saline and sonicated to homogenize and disrupt cell membranes. Cell homogenate was then measured for protein content by standard bicinchoninic acid assay and followed by Folch extraction. Internal standards were added to quantify phospholipids. After overnight incubation at 4°C, the bottom layer of the lipid phase was dried under nitrogen gas and redissolved with chloroform:isooctane (1:1) and transferred to HPLC vial for further analysis. The proportion of PC and phosphatidylethanolamine (PE) was determined, and PC:PE ratio was calculated.

### PBMC Phenotype Analysis

Two to four multicolor flow cytometry panels were designed and PBMCs were stained with different antibodies to the following markers: T cell panel: CD3 (PerCP), CD4 (FITC), CD4 (PerCP), CD8 (PE), CD25 (STAR PE), CD80 (APC), and CD45RA (PE); Antigen-presenting cell (APC) panel: CD14 (FITC), CD11c (PE), CD284 (AF647), SLAII (FITC) and CD21 (PE). Briefly, 100 μl of whole blood was added to pre-treated 96 well plates and incubated twice with lysis buffer. Lysed cells were then washed with 5% fetal calf serum in phosphate-buffered saline and spun down. Cells were then stained with fluorescently tagged antibodies (all purchased from BD Biosciences, BioLegend, Bio-Rad Laborarories, USA) at 4°C for 30 min, followed by a wash with phosphate-buffered saline and fixation in paraformaldehyde (10 g/l, Thermo Fisher Scientific) until further analysis. All samples were acquired within 72 h of preparation by BD LSR Fortessa flow cytometer at University of Alberta and data was analyzed using FlowJo v10 (USA).

### *Ex vivo* Cytokine Production From Mitogen Stimulated PBMCs

Isolated PBMCs (1.25 ×10^6^ cells/ml) were cultured without mitogens (unstimulated) or with mitogens: phorbol myristate acetate-ionomycin (PMA-I, T cell mitogen, Fisher Scientific, 2 2 μg/ml), phytohemagglutinin (PHA, T cell mitogen, Sigma-Aldrich, 25 μg/ml), pokeweed mitogen (PWM, T and antigen presenting cells mitogen, Sigma-Aldrich, 55 μg/ml). After 48 h incubation, samples were centrifuged at 1,500 rpm for 10 min to pellet cells. Supernatant was collected and stored at −80°C. Concentrations of interleukin (IL)-1β, IL-2, IL-6, IL-10, IFN-γ, and TNF-α were measured by commercial porcine specific ELISA kits (R&D systems, Minnesota) following manufacturer's instructions. Concentrations of cytokines produced from PBMCs were quantified on a microplate reader (wavelength at 450 nm to 570 nm, SpectraMax 190, Molecular Devices). Samples were assayed in duplicate with CV < 10%.

### Statistical Analyses

All statistics were performed by using one-way ANOVA procedure with Tukey adjustment for multiple comparisons between groups (Prism 8). All tests and comparisons were considered significant at *P*-value <0.05. Based on our previous study using diet induced swine model of insulin resistance, we have been able to detect 20% differences on average with a 95% CI with *n* = 4–5 piglets per group when comparing BW differences (15–20% difference; LBW vs. NBW) and diet induced differences (20–30% difference; CHF vs. Chow) on fasting lipid profile, insulin and glucose. As this was our first time assessing immune function in this pre-established model, we aimed to increase our sample size to *n* = 8 piglets per group in order to detect significant differences in immune outcomes. All results were expressed as means ± standard error mean (SEM) unless otherwise stated.

## Results

### Pig Growth and Daily Food Intake Parameters

As expected, the birthweights of piglets in all three LBW groups were lower than piglets in NBW groups (all, *P* < 0.05, Supplementary Table 1). The NBW-Chow group had a lower average energy intake compared to all high-fat diet fed groups (all, *P* < 0.05). The energy to weight gain ratio of NBW-Chow piglets were also lower than all other groups (all, *P* < 0.001); however, there were no differences in average daily growth. There were also no differences on final bodyweight among all NBW and LBW groups. LBW piglets caught up to the NBW piglets starting from 5 weeks of age as shown in Supplementary Figure 1.

### Fatty Acid Profile in Liver Tissue

The analysis was conducted in all LBW groups only, as CHF is considered the control group for comparing the effect of dairy in this model and are presented in Supplementary Table 2. In liver tissue, the proportion of myristic acid (C14:0) was higher in LBW-HFDairy compared to both LBW-CHF and LBW-LFDairy (both *P* = 0.001). Similarly, the unique dairy-derived odd-chain fatty acid, pentadecanoic acid (C15:0), was also higher in LBW-HFDairy compared to both LBW-CHF and LBW-LFDairy (both *P* < 0.004). The proportion of eicosapentaenoic acid (EPA, C20:5 n3) was higher in LBW-CHF compared to LBW-HFDairy only (*P* < 0.01). There were no differences in the proportion of total SFA, mono- (MUFA) and polyunsaturated fatty acids (PUFA) across all LBW groups.

### Fasting Plasma Lipids, Glucose, Insulin, HOMA-IR and Inflammatory Marker Profiles

**Figure 2:**
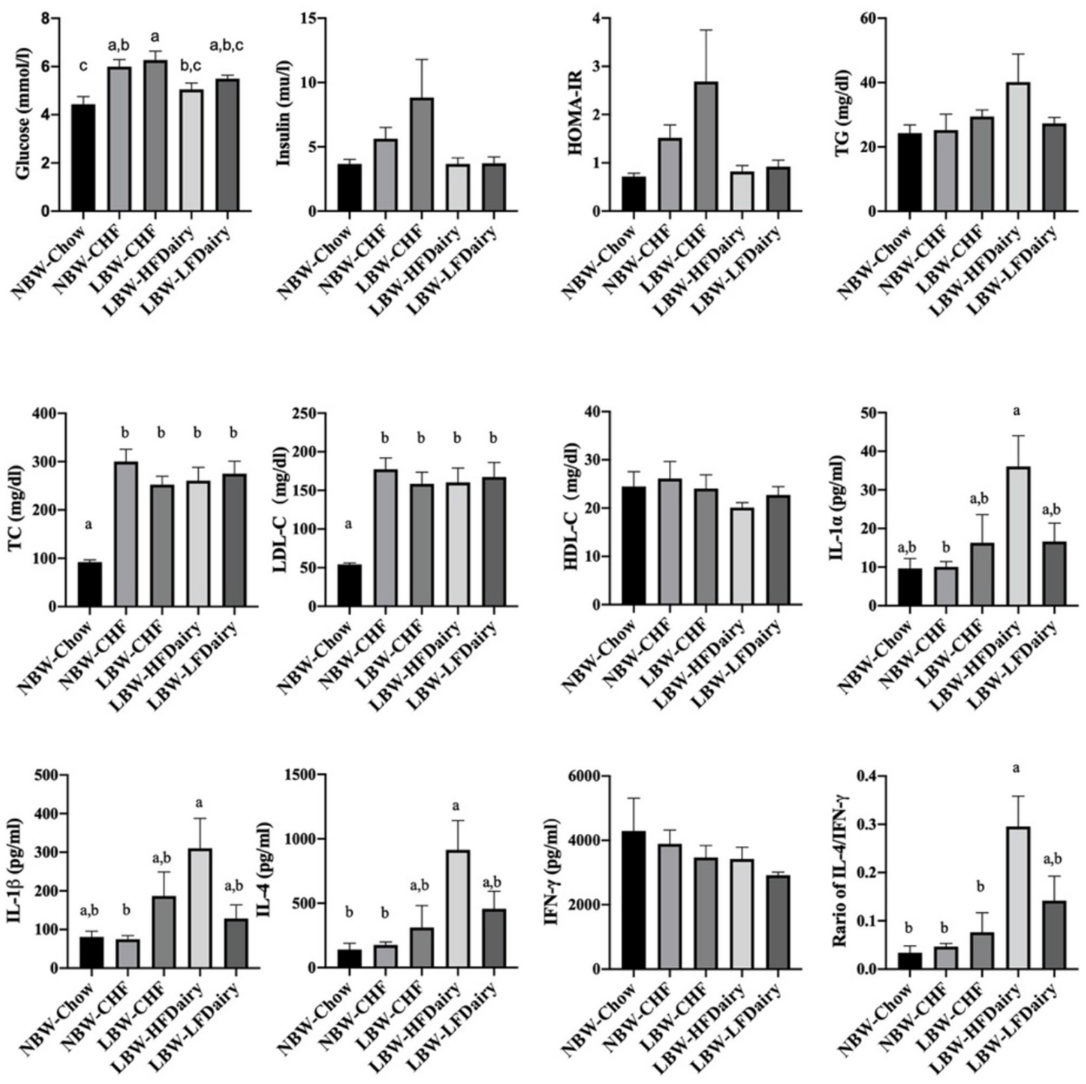
Changes on fasting plasma biochemical parameters and inflammatory cytokines in LBW and NBW swine fed different experimental diets. *P*-model for insulin = 0.094; *P*-model for HOMA-IR = 0.08. CHF, control high-fat diet; HDL-C, high-density lipoprotein cholesterol; HFDairy, high-fat dairy diet; HOMA-IR, homeostatic model assessment for insulin resistance; IFN-γ, interferon-gamma; IL, interleukin; LBW, low birthweight; LDL-C, low-density lipoprotein cholesterol; LFDairy, low-fat dairy diet; NBW, normal birthweight; TC, total cholesterol; TG, triglycerides; TNF-α, tumor necrosis factor-alpha. All values are expressed as means ± SEM. A *p*-value was considered as statistically significant when <0.05. Means sharing the same letter were not significantly different from each other.

Fasting plasma lipid profile, glucose, insulin and calculated HOMA-IR are shown in Figure 2. Fasting glucose in NBW-Chow was lower than in NBW-CHF and LBW-CHF (both *P* < 0.01). The LBW-HFDairy had lower plasma glucose concentrations compared to the LBW-CHF group (*P* < 0.05). Feeding low-fat dairy products also improved (lowered) fasting glucose concentrations in that they were no longer different to NBW-Chow, but to a lesser extent than high-fat dairy products. Although no statistical differences were observed in fasting insulin concentrations and HOMA-IR among all groups, there was a trend toward higher HOMA-IR in the LBW-CHF group when compared to all other groups (*P*-model = 0.08) suggesting early signs of insulin resistance.

**Figure 3:**
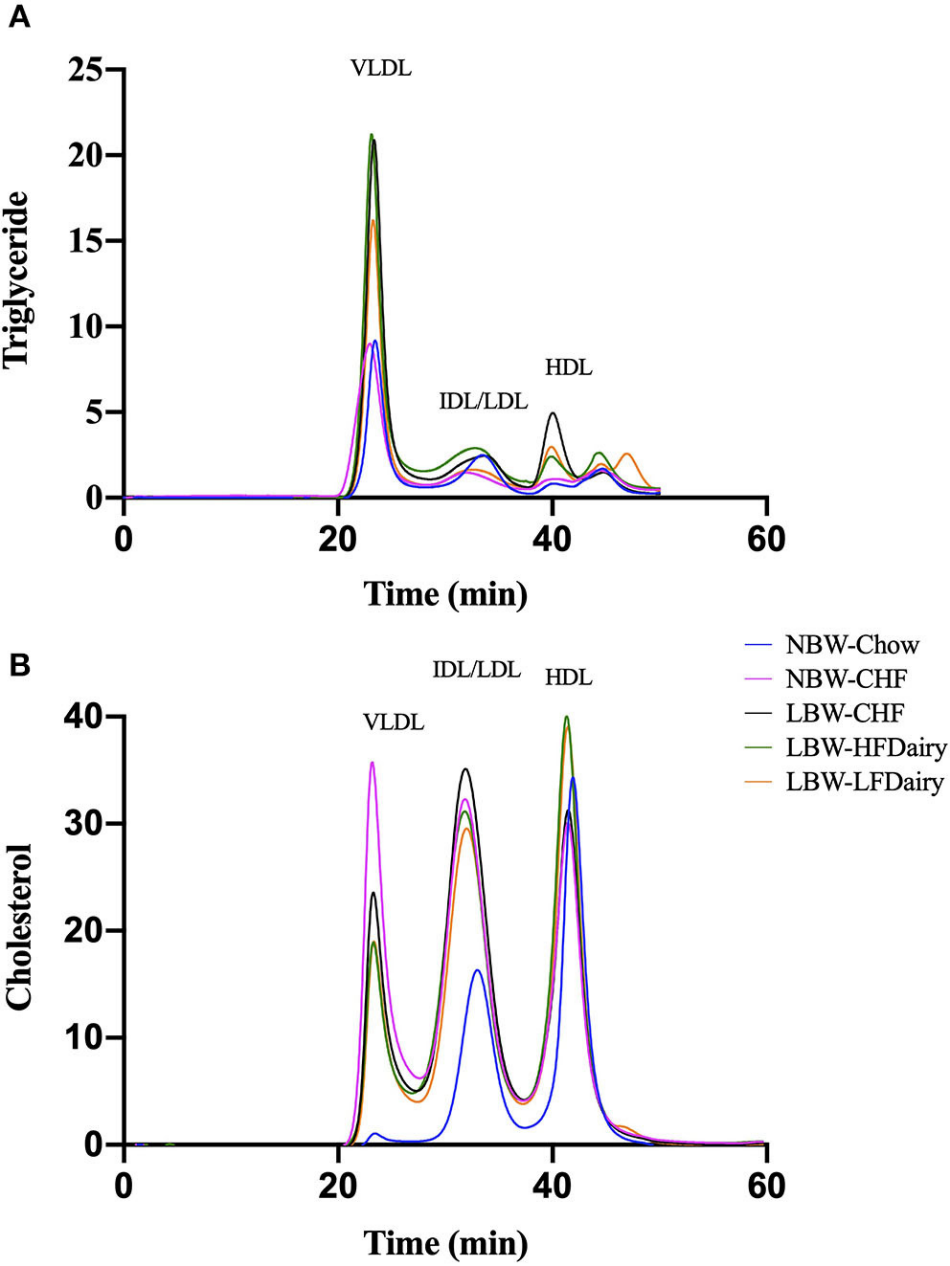
Fasting plasma triglyceride **(A)** and cholesterol **(B)** in lipoprotein subfractions measured by FPLC. CHF, control high-fat diet; FPLC, fast protein liquid chromatography; HDL, high-density lipoprotein; HFDairy, high-fat dairy diet; IDL, intermediate-density lipoprotein; LBW, low birthweight; LDL, low-density lipoprotein; LFDairy, low-fat dairy diet; NBW, normal birthweight; VLDL, very low-density lipoprotein.

There were no differences in plasma TG and HDL-C concentrations across all groups. However, plasma TC concentrations in NBW-Chow were lower than in all other high-fat diet fed groups (all, *P* < 0.001). Similarly, LDL-C concentrations in NBW-Chow were lower compared to all other groups (all, *P* < 0.05). Consistent with this, FPLC results showed that the NBW-Chow had the lowest amount of cholesterol in very low-density lipoprotein (VLDL), intermediate density lipoprotein (IDL) and LDL particles compared to all other high-fat diet fed groups (Figure 3). Although no significant difference was observed in fasting plasma TG levels among groups, the three LBW groups had increased TG in VLDL particles compared to both NBW groups.

Full results on fasting circulating inflammatory marker concentrations are shown in Supplementary Table 3. IL-1α and IL-1β in LBW-HFDairy were higher than in NBW-CHF (both *P* < 0.05). IL-4 in LBW-HFDairy was higher than in both NBW-Chow and NBW-CHF (both *P* < 0.05). The ratio of IL-4/IFN-γ in LBW-HFDairy was higher than in NBW-Chow, NBW-CHF and LBW-CHF (Figure 2, all, *P* < 0.05). There were no differences in IL-1Ra, IL-2, IL-6, IL-8, IL-10, IL-12. IL-18, IFN-γ, and TNF-α concentrations across all the groups.

### PBMC Membrane Phospholipid Classes

**Figure 4:**
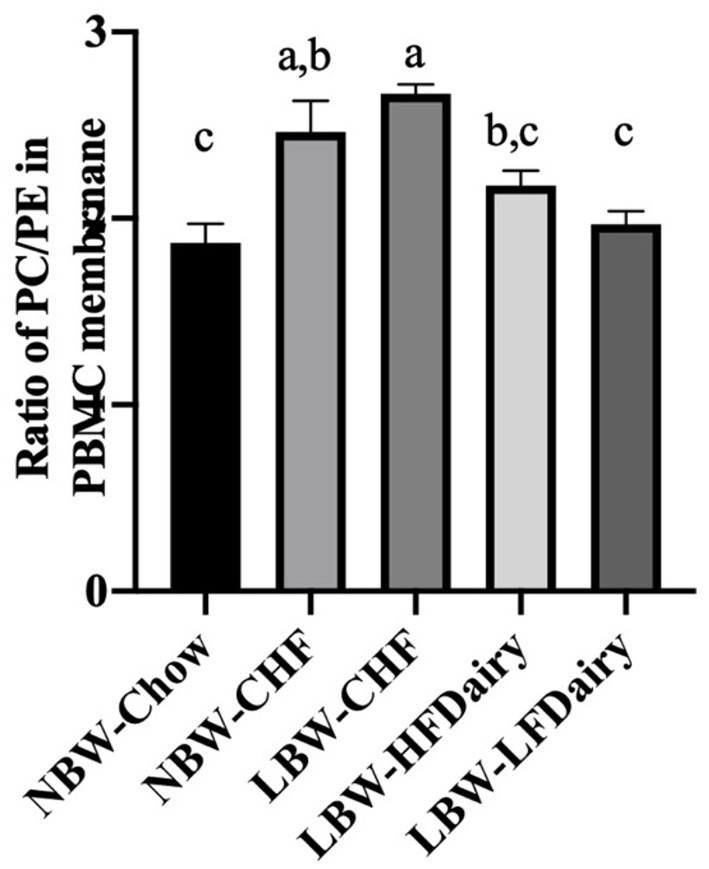
The phosphatidylcholine to phosphatidylethanolamine ratio in PBMC membrane. CHF, control high-fat diet; HFDairy, high-fat dairy diet; LBW, low birthweight; LFDairy, low-fat dairy diet; NBW, normal birthweight; PBMC, peripheral blood mononuclear cell; PC, phosphatidylcholine; PE, phosphatidylethanolamine. All values are expressed as means ± SEM. A *p*-value was considered as statistically significant when <0.05. Means sharing the same letter were not significantly different from each other.

No differences were observed in the total amount of the different phospholipid classes in PBMC membranes except for PE and PC. PE and PC were found to be higher and lower, respectively, in the NBW-Chow and LBW-LFDairy groups compared to the LBW-CHF group (both *P* < 0.05, data not shown). In Figure 4, the ratio of PC:PE was found to be lower in NBW-Chow and LFDairy groups compared with both the NBW-CHF and LBW-CHF groups (*P* < 0.001).

### *Ex vivo* Cytokine Production From Mitogen Stimulated PBMCs

*Ex vivo* cytokine production by PBMCs stimulated with mitogens is presented in Table 2. Following PWM stimulation, IL-2, TNF-α and IFN-γ production were significantly lower in LBW-CHF than in NBW-Chow (all, *P* < 0.001). While feeding high-fat dairy products had minimal effect on T helper cell (Th) 1 cytokine production, feeding low-fat dairy products significantly improved IL-2, TNF-α and IFN-γ production compared to LBW-CHF (all, *P* < 0.05). Additionally, LBW-CHF piglets were also found to produce less IL-1β compared to NBW-Chow (*P* < 0.01) and less IL-10 compared to both NBW-Chow (*P* < 0.001) and NBW-CHF (*P* < 0.001). Dairy products normalized the IL-1β production in that it was no longer different from the NBW-Chow group but did not normalize IL-10 production.

**Table 2:** *Ex vivo* cytokine production by mitogen stimulated PBMCs in LBW and NBW swine fed different experimental diets.

	**NBW-Chow**	**NBW-CHF**	**LBW-CHF**	**LBW-HFDairy**	**LBW-LFDairy**	***P***
PWM (T and APC cell mitogen)						
IL-2, pg/ml	494.7 ± 76.6^a^	297.0 ± 39.8^b, c^	130.7 ± 26.0^c^	148.9 ± 16.1^c^	313.8 ± 55.5^a, b^	<0.001
TNF-α, pg/ml	1,385 ± 195.5^a^	567.8 ± 57.8^c^	686.3 ± 82.2^c^	691.3 ± 56.1^c^	1,105 ± 73.2^a^	<0.001
IFN-γ, pg/ml	494.7 ± 76.6^a^	297.0 ± 39.8^b, c^	130.7 ± 26.0^c^	148.9 ± 16.1^c^	313.8 ± 55.5^a, b^	<0.001
IL-1β, pg/ml	3,754 ± 348.3^a^	2187 ± 136.8^a, b^	2,125 ± 306.1^b^	2,960 ± 292.5^a, b^	2,918 ± 139.1^a, b^	0.007
IL-6, pg/ml	258.3 ± 51.5	156.9 ± 62.4	161.6 ± 38.5	214.4 ± 27.3	163.9 ± 28.6	0.411
IL-10, pg/ml	3,014 ± 428.1^a^	2,455 ± 194.2^a^	1,121 ± 103.0^b^	998.4 ± 102.4^b^	1,622 ± 167.9^b^	<0.001
PHA (T cell mitogen)						
IL-2, pg/ml	234.0 ± 25.2^a^	227.4 ± 28.6^a^	116.3 ± 12.5^b^	176.5 ± 15.3^a, b^	220.3 ± 20.3^a^	0.002
TNF-α, pg/ml	365.1 ± 61.9	378.3 ± 40.6	232.2 ± 45.4	242.2 ± 26.9	370.5 ± 41.6	0.031
IFN-γ, pg/ml	77.6 ± 17.7^a, b^	52.3 ± 15.1^a, b^	37.7 ± 10.2^b^	29.3 ± 5.5^b^	102.2 ± 19.7^a^	0.006
IL-6, pg/ml	160.2 ± 21.3	121.7 ± 25.7	93.9 ± 40.8	94.4 ± 21.4	190.5 ± 24.2	0.059
IL-10, pg/ml	2,422 ± 448.3^a^	1,693 ± 235.5^a, b^	1,052 ± 183.0^b^	958.3 ± 137.7^b^	1,326 ± 171.8^b^	0.001
PMA-I (T cell mitogen)						
IL-2, pg/ml	1,343 ± 282.4	1,675 ± 184.1	1,056 ± 197.4	1,685 ± 195.6	1,686 ± 207.2	0.138
TNF-α, pg/ml	3,611 ± 882.4^a, b^	3,242 ± 229.4^a, b^	1,915 ± 250.5^b^	3,514 ± 357.5^a, b^	4,139 ± 437.1^a^	0.013
IFN-γ, pg/ml	4,207 ± 491.4	3,587 ± 152.2	2,366 ± 744.0	3,537 ± 657.5	4,383 ± 261.3	0.178
IL-6, pg/ml	615.1 ± 167.0^a^	279.6 ± 66.8^a, b^	147.9 ± 47.8^b^	114.2 ± 30.2^b^	237.4 ± 45.5^b^	0.009
IL-10, pg/ml	2,185 ± 437.8^a^	2,035 ± 84.8^a, b^	1,095 ± 181.8^b^	1,319 ± 142.6^a, b^	1,652 ± 152.3^a, b^	0.012

*APC, antigen presenting cell; CHF, control high-fat diet; HFDairy, high-fat dairy diet; IFN-γ, interferon-gamma; IL, interleukin; LBW, low birthweight; LFDairy, low-fat dairy diet; NBW, normal birthweight; PBMC, peripheral blood mononuclear cell; PHA, phytohemagglutinin; PMA-I, phorbol 12-myristate 13-acetate plus ionomycin; PWM, pokeweed mitogen; TNF-α, tumor necrosis factor-alpha. All values are expressed as means ± SEM. A p-value was considered as statistically significant when <0.05. Means sharing the same letter were not significantly different from each other*.

Following PHA stimulation, IL-2 production was significantly lower in LBW-CHF than in NBW-Chow and NBW-CHF (all, *P* < 0.05). While Feeding high-fat dairy products had minimal effects on IL-2 production, feeding low-fat dairy products significantly improved IL-2 production compared to LBW-CHF (*P* < 0.01). TNF-α and IFN-γ production also tended to be lower in LBW-CHF than in NBW-Chow and NBW-CHF (all, *P* model <0.05). Similarly, while feeding high-fat dairy products had minimal effects on TNF-α and IFN-γ production, feeding low-fat dairy products significantly improved IFN-γ production compared to LBW-CHF (*P* < 0.05). Additionally, LBW-CHF was found to produce less IL-10 compared to NBW-Chow (*P* < 0.01); however, dairy products had minimal effect.

Following PMA-I stimulation, there was an effect of treatment (*P* model <0.05) on TNF-α production and tended to be lower in LBW-CHF than in NBW-Chow and NBW-CHF. While feeding high-fat dairy products had minimal effect on TNF-α production, feeding low-fat dairy products significantly improved TNF-α production compared to LBW-CHF (*P* < 0.01). IL-6 and IL-10 production were also lower in LBW-CHF than in NBW-Chow (all, *P* < 0.05), and the production of IL-10 was no longer different compared to NBW-Chow in both LBW-HFDairy and LBW-LFDairy groups.

### PBMC Immune Cell Phenotypes

As presented in Table 3, the proportion of T cells (CD3+) were similar among all groups except for the LBW-LFDairy, which had a slightly higher proportion compared to the NBW-CHF (*P* < 0.05). No change in the overall proportion of helper T cells and cytotoxic T cells were observed among groups. No other differences were observed among groups for any other T cell markers including naïve T cells (CD3+CD45RA+) and helper and cytotoxic T cells expressing the IL-2 receptor (CD3+CD4+CD25+ and CD3+CD8+CD25+). We were unable to precisely identify B cell population (CD21+) due to the insufficient antibody staining and therefore, results not shown.

**Table 3:** PBMCs population of NBW and LBW swine fed different experimental diets.

**Phenotype**	**NBW-Chow**	**NBW-CHF**	**LBW-CHF**	**LBW-HFDairy**	**LBW-LFDairy**	***P***
% of gated cells						
Total CD3+ (T cell)	61.3 ± 4.1^a, b^	59.6 ± 1.7^b^	63.3 ± 1.7^a, b^	65.5 ± 2.3^a, b^	69.5 ± 1.2^a^	0.032
CD3+CD4+ (Th cell)	62.4 ± 2.4	61.0 ± 2.9	64.8 ± 2.0	59.3 ± 2.9	59.2 ± 2.5	0.519
CD3+CD8+ (cytotoxic T cell)	37.7 ± 2.5	38.8 ± 2.9	34.4 ± 2.4	40.6 ± 2.9	41.2 ± 2.5	0.383
CD3+CD45RA+ (naïve T cell)	16.2 ± 2.4	16.9 ± 0.6	18.4 ± 1.8	14.1 ± 2.7	16.0 ± 2.4	0.712
Total CD25+ (IL-2 receptor)	28.2 ± 1.3	29.4 ± 2.8	29.8 ± 1.9	29.5 ± 1.3	26.2 ± 2.5	0.676
CD3+CD4+CD25+	30.5 ± 1.1	28.2 ± 4.2	36.1 ± 4.5	40.0 ± 4.9	30.0 ± 3.7	0.257
CD3+CD8+CD25+	22.2 ± 2.0	20.7 ± 2.2	20.1 ± 0.7	20.8 ± 1.4	19.7 ± 1.9	0.875
Total CD80+ (co-stimulator of T cell)	14.6 ± 0.9	10.0 ± 1.2	8.3 ± 2.2	8.1 ± 1.5	8.8 ± 1.4	0.207
Total CD14+	7.2 ± 0.7^a^	6.5 ± 1.1^a^	5.4 ± 0.7^a, b^	2.8 ± 0.3^c^	3.5 ± 0.5^b, c^	<0.001
CD14+CD11c+	8.7 ± 2.4	8.2 ± 3.4	7.7 ± 2.3	5.9 ± 2.1	3.6 ± 1.1	0.502
Total CD284+ (TLR-4)	3.9 ± 0.4^a^	3.0 ± 1.1^a, b^	1.2 ± 0.3^b^	1.3 ± 0.4^b^	2.5 ± 0.6^a, b^	0.021
CD14+CD284+	25.2 ± 3.6^a^	11.6 ± 4.8^b^	9.8 ± 2.5^b^	7.3 ± 1.3^b^	9.8 ± 1.6^b^	0.002
SLAII+CD284+	11.0 ± 0.8^a^	2.9 ± 0.7^b^	2.7 ± 0.8^b^	2.2 ± 0.7^b^	3.0 ± 0.8^b^	<0.001

*B cell population (CD21+) was not shown due to the insufficient antibody staining; CHF, control high-fat diet; HFDairy, high-fat dairy diet; IL, interleukin; LBW, low birthweight; LFDairy, low-fat dairy diet; NBW, normal birthweight; PBMC, peripheral blood mononuclear cell; Th, T helper cell; TLR-4, toll-like receptor 4. All values are expressed as means ± SEM. A p-value was considered as statistically significant when <0.05. Means sharing the same letter were not significantly different from each other*.

The proportion of monocytes (CD14+) was lower in LBW-HFDairy compared to NBW-Chow, NBW-CHF and LBW-CHF, and was lower in LBW-LFDairy compared to NBW-Chow and NBW-CHF (all, *P* < 0.05). No differences were observed among groups for activated monocytes (CD14+CD11c+). The proportion of APCs expressing toll-like receptor 4 (TLR-4, CD284+) was lower in both the LBW-CHF and LBW-HFDairy but not in the LBW-LFDairy compared with the NBW-Chow (all, *P* < 0.05). However, the proportion of monocytes expressing the TLR-4 (CD14+CD284+) and APCs expressing both major histocompatibility complex (MHC) class II and TLR-4 (SLAII+CD284+) were lower in all high-fat diet fed groups than in NBW-Chow (all, *P* < 0.05).

## Discussion

**Figure 5:**
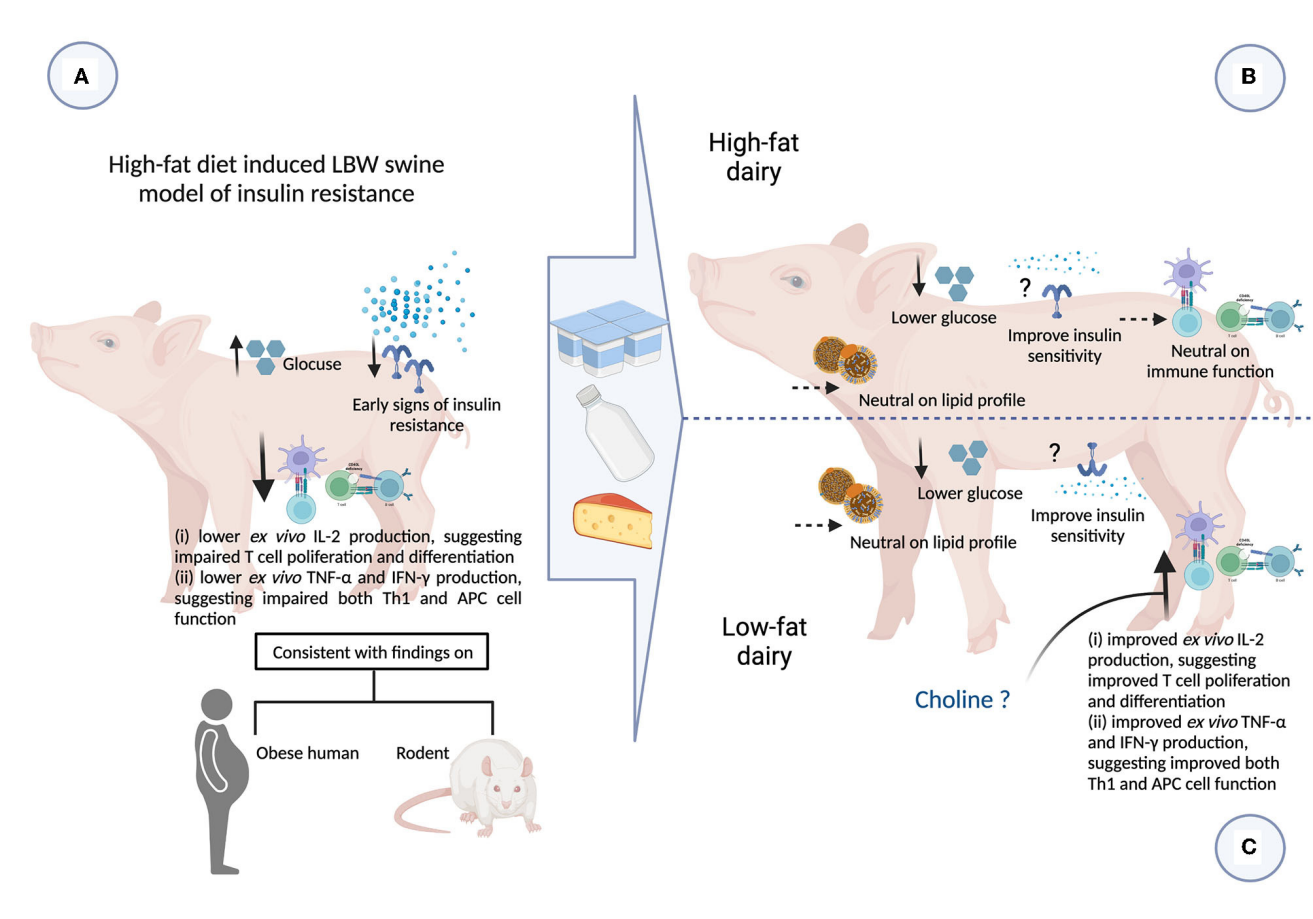
Summary of impact of consuming 3 servings high- and low-fat dairy products on obesity-related cardiometabolic and immune perturbations (figure created with BioRender). APC, antigen presenting cell; CHF, control high-fat diet; HFDairy, high-fat dairy diet; IFN-γ, interferon-gamma; IL, interleukin; LBW, low birthweight; LDL-C, low-density lipoprotein cholesterol; LFDairy, low-fat dairy diet; NBW, normal birthweight; TC, total cholesterol; Th, T helper cell; TG, triglycerides; TNF-α, tumor necrosis factor-alpha. **(A)** LBW piglets fed with a high-fat diet (LBW-CHF) developed elevated fasting glucose concentrations and early signs of insulin resistance. Following *ex vivo* immune cell stimulation, poor IL-2, TNF-α and IFN-γ productions was observed suggesting impaired T and APC cell function compared to NBW control groups. This is also consistent with findings in individuals with obesity and rodent models. **(B)** Feeding high-fat dairy products (LBW-HFDairy) improved (lowered) fasting glucose while had minor effects on immune function compared to LBW-CHF. **(C)** Feeding low-fat dairy products (LBW-LFDairy) also improved (lowered) fasting glucose while significantly improved IL-2, TNF-α, and IFN-γ productions following *ex vivo* stimulation, suggesting improved T and APC function compared to LBW-CHF. Feeding dairy products, regardless of fat content, had neutral effects on lipid profile.

In the present study we assessed the intake of 3 servings/day of either low- or high-fat commercially available dairy products, that included milk, yogurt and cheese, on obesity-related cardiometabolic perturbations and immune function. We have confirmed that LBW piglets fed a high-fat diet exhibited early signs of insulin resistance and also demonstrated peripheral immune dysfunction. Feeding dairy products, regardless of fat content, was associated with improvements in fasting plasma glucose levels. Feeding low-fat dairy products improved peripheral immune function to a greater extent than high-fat dairy. These findings provide novel insights into the role of dairy products and dairy fat in modulating obesity-related cardiometabolic perturbations and immune dysfunction (Figure 5).

### Dairy Fat and Its Effect on Choline Moieties in PBMC's Membrane and Systemic Inflammation

Intriguingly, our findings contradicted part of our initial hypothesis that high-fat dairy would potentially be more effective than low-fat dairy intake at improving immune function. This is likely in part due to the differences in choline content (and the forms of choline) between low- and high-fat dairy products. Unlike the structure of other dietary fats, milk fat droplets are normally enveloped by an amphiphilic membrane, namely milk fat globular membrane (MFGM). Polar lipids including PC, PE and SM are naturally found in dairy and specifically in the MFGM. During industrial processes such as making butter from raw milk, the MFGM together with polar lipids are disassociated from fat globules and preferentially enriched in the aqueous phase such as skimmed milk and buttermilk (28). Indeed, we have previously compared the choline content in 48 commercial dairy products in Canada and reported that total as well as both water- and lipid-soluble forms of choline were negatively associated with total dairy fat content (29). Our group has also recently demonstrated that buttermilk, as an important source of lipid soluble forms of choline (i.e., PC and SM), exhibited promising ability to support the immune system development and T cell function (16, 17). Therefore, the higher choline content in low-fat dairy products might explain, at least in part, the ameliorated immune function seen in the LBW-LFDairy group. We also assessed the two major phospholipid classes (PE and PC) in PBMCs membrane as an indirect marker of dietary PC intake. In rodents, we have previously shown that feeding a diet containing 100% PC as compared to a diet containing 100% free choline increased the PC content in splenocyte cell membranes which was associated with enhanced T cell function (30). Here, we demonstrated that the PC:PE ratio in PBMCs increases in the context of high-fat diet feeding, which can be normalized by dairy consumption with the most beneficial effect observed with low-fat dairy. Although there is limited evidence regarding the importance of the PC:PE ratio on immune function, a higher ratio has been linked to the progression of several metabolic diseases such as liver steatosis in *ob/ob* mice and insulin resistance in skeletal muscle (31). These data suggest that part of the greater beneficial effect that low-fat dairy has on immune function may be attributable to the higher amount of lipid soluble forms of choline normalizing the PC:PE ratio in the context of a high-fat diet.

The fact that high-fat dairy led to fewer changes in immune function than low-fat dairy in this model could also be due to the overall higher proportion of fat coming from the diet in this group. The proportion of fat coming from the 3 servings of high-fat dairy is roughly 64% whereas the proportion of fat in the low-fat dairy is about 26%. Therefore, by substituting some of the CHF diet that contains 46% fat by 3 servings of high-fat dairy, the overall proportion of fat in the diet was increased to roughly 51%. It has been demonstrated that high-fat diet and obesity can lead to an increased gut permeability and lipopolysaccharide (LPS) translocation which in turn would trigger an inflammatory response (32–34). Similarly, we demonstrated that feeding a high-fat diet in this swine model tended to increase circulating levels of inflammatory markers. Therefore, in the context of a high-fat diet, providing a diet that contains a higher proportion of fat may have led to a higher LPS translocation and greater systemic inflammation as observed in the LBW-HFDairy group. Indeed, we have observed higher concentrations of circulating IL-1α and IL-1β in the LBW-HFDairy group. In addition, LBW-HFDairy group also had a higher Th2 cytokine profile (reflected by the ratio of IL-4/IFN-γ), which is known to suppress Th1 responses. Collectively, this could explain, at least in part, the lower immune function observed in that group when compared to LBW-LFDairy.

### Dairy Fat and Its Impact on Glucose Metabolism and Insulin Resistance

Although the proportions of total SFA, MUFA and PUFA in liver tissue were similar among all LBW groups, the proportion of C14:0 and C15:0 were higher in the LBW-HFDairy group. These SFAs have been used as biomarkers of dairy fat intake in humans (20, 35–38). Particularly, C15:0 and C17:0, are odd-chain SFAs that cannot be endogenously synthesized and are specific to dairy and ruminant meat (37). However, it has been previously reported that these two odd-chain SFAs are present in a variety of marine fish species (39). Hence, it is not surprising that we have also detected C15:0 and C17:0 and higher proportion of EPA in liver tissue in the group that received the CHF diet since fish meal is added to the experimental diet. Both dairy groups consumed less of the experimental CHF diet to account for the energy intake coming from dairy consumption and therefore accumulated less EPA. However, we confirmed that the biomarkers for dairy fat intake in humans are also relevant for this large animal swine model.

Meta-analyses of cohort studies have previously reported an inverse relationship between biomarkers of dairy fat intake, particularly the C15:0 and C17:0, and the incident of T2D in humans (14, 40). *In vitro* studies have also reported that C15:0 play a direct role in glucose metabolism *via* promoting GLUT4 translocation to plasma membrane in myotubes (41). C15:0 has also recently been proposed to be an essential fatty acid due to a number of established cardiometabolic benefits including attenuation of glucose concentrations in mice fed with high-fat diet (13). Importantly, our findings in LBW swine model are in agreement with previous findings, suggesting that dairy fat may be responsible, at least to some extent, for the improvement in glucose metabolism in this model.

### Dairy Fat and Its Impact on Dyslipidemia

Dairy fat is still a subject of controversy as it relates to its beneficial effect on cardiometabolic health due to its high content in SFA. Yet, emerging evidence from randomized controlled trials (RCT) mostly point to a neutral effect of dairy, irrespective of their fat content, on several cardiometabolic risk factors (21). For instance, a recent RCT in 72 subjects with metabolic syndrome reported that consumption of 3.3 servings/day of either low- or high-fat dairy from the same food matrix, specifically milk, yogurt and cheese for 12 weeks, did not modulate serum concentrations of TC, LDL-C, HDL-C, and TG (42). We also reported a neutral effect of dairy consumption, regardless of their fat content, on TC, LDL-C, HDL-C and TG when compared to the LBW-CHF group. Altogether, our results suggest that feeding dairy products in the context of a high-fat diet has little effect on the lipid profile.

### Dairy and Their Impact on Peripheral Blood T Cell Function

Previous findings in humans and rodents have demonstrated an impaired immune response to mitogen stimulation in the context of obesity and T2D (7, 43, 44). Here, we found that consuming a high-fat diet in the context of a LBW swine model of insulin resistance also led to impaired immune function, particularly impaired T cell function. IL-2 is a cytokine that induces T cell proliferation and differentiation (45). Lower IL-2 production after PHA and PWM stimulation in the LBW-CHF suggests an impaired T cell proliferation and differentiation. Additionally, IFN-γ and TNF-α are two crucial cytokines produced by Th1 cells and in turn, also being a major element to induce T cell proliferation (46). Therefore, the lower IFN-γ and TNF-α production after PWM stimulation in LBW-CHF could explain, to some extent, the lower IL-2 production in this group. On the other hand, we have demonstrated that feeding low-fat dairy in the context of a high-fat diet exerted a greater ability to normalize IL-2, IFN-γ, and TNF-α production after stimulation relative to the NBW-Chow group, suggesting that low-fat dairy may be more effective than high-fat dairy. Remarkably, we still observed that feeding high-fat dairy improved IL-2 after PHA, and TNF-α after PMA-I stimulation even though it was to a lesser extent than the low-fat dairy. Previous studies in humans have reported that individuals with obesity and T2D [when compared to individuals with obesity but metabolically healthy (MHO)] have higher proportions of naïve T cells (CD3+CD45RA+) and cytotoxic T cells despite having similar proportions of total T cells (7). Moreover, individuals with obesity and T2D had a higher proportion of immune cells expressing activation markers such as CD80, and T cells expressing CD278, which play an important role in IL-2 production and T cell proliferation (7). In the current study, we did not observe any significant changes on the major T cell subsets or activation markers. A possible explanation is that although our model was characterized by early signs of insulin resistance, it did not lead to a state of frank T2D. Altogether, our data suggest that a high-fat diet impairs T cell function in LBW swine and that feeding low-fat dairy can counteract some of the obesity-related T cell dysfunction.

### Dairy and Their Impact on Peripheral Blood APC Function

IFN-γ and TNF-α can also be produced by APCs such as monocytes and dendritic cells. IL-1β is another crucial cytokine that can be induced by nearly all microbial substances and causes inflammation (47). Therefore, lower levels of IFN-γ, TNF-α, and IL-1β produced from LBW-CHF after PWM stimulation may also suggest an impaired innate immune response. Similarly, feeding low-fat dairy normalized the production of these cytokines in that they were no longer differ from the NBW-Chow. Feeding high-fat dairy also normalized the production of IL-1β, but not the other cytokines measured. This could be explained, at least in part, by the differences in total cells expressing CD284+ (TLR-4) in PBMCs. TLR-4 is known to be one of the first lines of defense for the innate immune system by recognizing bacterial endotoxins and inducing subsequent immune responses (48). Indeed, we reported that the expression of TLR-4 was lower in both the LBW-CHF and LBW-HFDairy groups while the LBW-LFDairy was similar to NBW-Chow. Decreased expression of TLR-4 has been previously reported in elderly patients who have a higher prevalence of infection than younger patients (49). In contrast, B cell proportions were previously found to be similar in individuals with obesity and T2D compared to MHO (7). This is consistent with the current finding that the proportions of B cells and macrophages remained unchanged across all groups. Overall, our data suggest that a high-fat diet may impair APCs function in LBW swine and that feeding low-fat dairy can improve APC function by partially normalizing the expression of TLR-4.

As much as the acute inflammatory response to stimuli is important in mounting an adequate immune response, the resolution phase of inflammation is as important to prevent tissue damage. IL-10 is a key regulatory cytokine with anti-inflammatory properties and can be produced by a number of myeloid and lymphoid cells including Th2, regulatory T cells (Treg), dendritic cells and macrophages (50). The action of IL-10 on promoting the resolution phase of inflammation has also been previously reported (51). In the present study, lower production of IL-10 in LBW-CHF after both T cell mitogen and APC mitogen stimulations is suggestive of an impaired anti-inflammatory cytokine production and overall diminished immune-suppressive ability. Indeed, serum concentrations of IL-10 have been found to be significantly reduced in obese subjects and correlated with hyperinsulinemia and insulin resistance (52). IL-10 has also been found to be less effective at inhibiting inflammation in T2D patients, and failed to attenuate TNF-α production upon LPS stimulation (53). On the other hand, we demonstrated that feeding dairy, regardless of fat content, normalized to some extent the IL-10 production after PMA-I stimulation. Although this suggests that dairy may improve the resolution phase of inflammation, the enhanced IL-10 production cannot be attributable to a specific cell type (i.e., APCs, Th2 or Treg cells) nor inflammatory phase. Thus, future studies are warranted to validate which cell type is responsible for the IL-10 production post-stimulation with more detailed laboratory assessment such as intra-cellular cytokine staining.

### Strength and Limitations

To the best of our knowledge, this is the first study using a swine model of obesity and insulin resistance to investigate the effect of low- vs. high-fat dairy products using similar food matrices on cardiometabolic risk factors and immune function. This is important since most studies in the field trying to understand the effect of dairy fat have compared milk or yogurt to cheese and butter which do not take into consideration the importance of the food matrix. The higher-than-expected inter-individual variations in some outcomes (i.e., insulin) suggest that future studies should increase the sample size and perhaps the duration of the intervention to induce a stronger insulin-resistant phenotype. As in any nutritional study, there is a replacement effect in that by adding dairy products to the diet, other nutrients were consumed in lower amounts. In this study, we aimed to match the overall energy intake among the high-fat diet treatment groups to compare the effect of adding dairy and dairy fat *per se* in the context of an isoenergetic diet. However, the high-fat dairy products contained more fat and slightly less protein compared to the low-fat dairy products, which led to an overall higher fat intake and a slight reciprocal reduction in protein and carbohydrate intake. Therefore, it is virtually impossible to attribute the effect observed in our study solely to adding dairy products. We have to stress however, that this type of nutritional study also reflects the real-life scenario in humans. Indeed, people consume whole foods and not isolated nutrients. Therefore, when incorporating dairy in our diet, most people will reduce their intake of other foods leading to differences in protein, carbohydrate and fat intake.

## Conclusion

In conclusion, findings from the present study provide new mechanistic evidence that support the role of dairy products, specifically milk, yogurt and cheese, in counteracting some of the cardiometabolic and immune dysfunction associated with obesity. Consumption of 3 servings per day of high-fat dairy products lowered fasting glucose more than low-fat dairy, whereas low-fat dairy improved immune function particularly T cell function to a greater extent than high-fat dairy. Irrespective of fat content, consumption of 3 servings of dairy products had a neutral effect on the lipid profile in this swine model of insulin resistance.

## Data Availability Statement

The original contributions presented in the study are included in the article/Supplementary Material, further inquiries can be directed to the corresponding author/s.

## Ethics Statement

The animal study was reviewed and approved by University of Alberta Animal Care and Use Committee.

## Author Contributions

CR and SP designed and obtained funding for this study. ST and BW provided expertise on immunology and animal model. YS, AM, RM, and KW conducted research and analyzed data. YS performed the statistical analysis and wrote the manuscript under the supervision of CR and SP. CR has primary responsibility for final content. All authors have read and approved the final manuscript.

## Funding

This study was supported by grants from Dairy Farmers of Canada (RES0042193) and the Natural Sciences and Engineering Research Council of Canada (RES0038933). YS is a recipient of a Ph.D. scholarship from China Scholarship Council (201807980001).

## Conflict of Interest

The authors declare that the research was conducted in the absence of any commercial or financial relationships that could be construed as a potential conflict of interest.

## Publisher's Note

All claims expressed in this article are solely those of the authors and do not necessarily represent those of their affiliated organizations, or those of the publisher, the editors and the reviewers. Any product that may be evaluated in this article, or claim that may be made by its manufacturer, is not guaranteed or endorsed by the publisher.

## Abbreviations

APC: antigen presenting cell
CHF: control high-fat diet
CI: confidence interval
CV: coefficient of variation
CVD: cardiovascular disease
ELISA: enzyme-linked immunosorbent assay
EPA: eicosapentaenoic acid
FPLC: fast protein liquid chromatography
GLUT4: glucose transporter type 4
HDL-C: high-density lipoprotein cholesterol
HFDairy: high-fat dairy diet
HOMA-IR: homeostatic model assessment for insulin resistance
HPLC: high performance liquid chromatography
IDL: intermediate-density lipoprotein
IFN-γ: interferon-gamma
IL: interleukin
LBW: low birthweight
LDL-C: low-density lipoprotein cholesterol
LFDairy: low-fat dairy diet
LPS: lipopolysaccharide
MF: milk fat
MFGM: milk fat globular membrane
MHC: major histocompatibility complex
MHO: metabolically healthy obese
MUFA: monounsaturated fatty acid
NBW: normal birthweight
PBMC: peripheral blood mononuclear cell
PC: phosphatidylcholine
PE: phosphatidylethanolamine
PHA: phytohemagglutinin
PMA-I: phorbol 12-myristate 13-acetate plus ionomycin
PUFA: polyunsaturated fatty acid
PWM: pokeweed mitogen
SEM: standard error of the mean
SFA: saturated fatty acid
SM: sphingomyelin
SRTC: swine research technology center
T2D: type-2 diabetes
TC: total cholesterol
TG: triglycerides
Th: T helper cell
TLR-4: toll-like receptor 4
TNF-α: tumor necrosis factor-alpha
Treg: T regulatory cell
VLDL: very low-density lipoprotein.
