# Supplementary material for: Effect of High-Fat and Low-Fat Dairy Products on Cardiometabolic Risk Factors and Immune Function in a Low Birthweight Swine Model of Diet-Induced Insulin Resistance
Source: Front Nutr. 2022 Jun 17;9:923120. doi: 10.3389/fnut.2022.923120 (PMC9247580; doi:10.3389/fnut.2022.923120)
Supplement: Supplementary file 1 [file Data_Sheet_1.pdf]

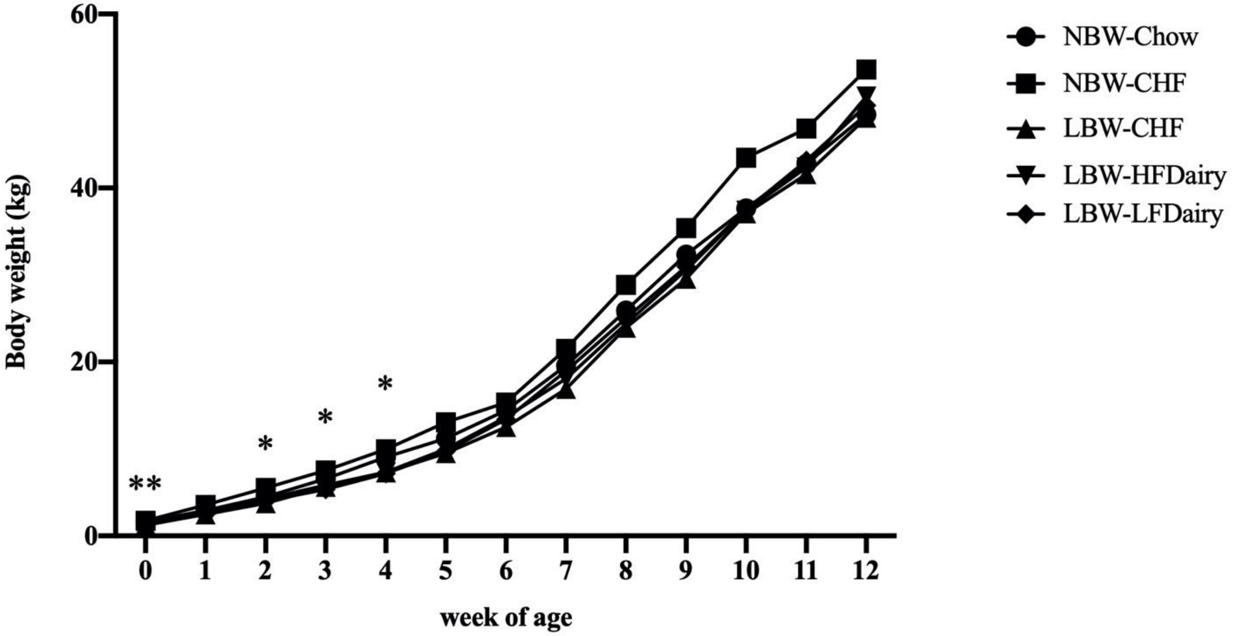

Supplementary Figure 1. Pig growth parameters during the intervention for NBW and LBW piglets fed the experimental diets. CHF, control high-fat diet; HFDairy, high-fat dairy diet; LBW, low birthweight; LFDairy, low-fat dairy diet; NBW, normal birthweight. A *p* value was considered as statistically significant when < 0.05. ** $P < 0.001$, *$P < 0.05$. At birth (week 0), body weight of all LBW groups were lower than NBW groups. At 2 weeks of age, body weight of LBW-CHF was lower than NBW-CHF. At 3 weeks of age, body weight of LBW-CHF and LBW-LFDairy were lower than NBW-CHF. At 4 weeks of age, body weight of all LBW groups were lower than NBW-CHF. Starting at the 5 weeks of age and until 12 weeks of age, there were no difference in body weight among groups.

Supplementary Table 1. Growth and feed consumption of LBW and NBW swine fed different experimental diets

	NBW-Chow	NBW-CHF	LBW-CHF	LBW-HFDairy	LBW-LFDairy	*P*
Birthweight (kg)	1.62±0.07[a]	1.73±0.10[a]	1.29±0.08[b]	1.23±0.06[b]	1.23±0.09[b]	<0.001
Final body weight (kg)	48.44±3.16	53.62±1.97	48.10±2.77	50.53±2.81	49.49±1.88	0.618
Average daily growth (g/day)	779.20±47.34	854.00±15.80	794.70±41.00	824.10±40.47	815.40±27.78	0.723
Average energy intake (Kcal/day)	4077±290.10[a]	5717±340.30[b]	5314±237.90[b]	5716±250.60[b]	5678±172.20[b]	0.001
Energy to gain ratio	5.23±0.14[a]	6.67±0.29[b]	6.72±0.15[b]	6.96±0.14[b]	6.97±0.10[b]	<0.001

CHF, control high-fat diet; HFDairy, high-fat dairy diet; LBW, low birthweight; LFDairy, low-fat dairy diet; NBW, normal birthweight. All values are expressed as means ± SEM. A p value was considered as statistically significant when <0.05. Means sharing the same letter were not significantly different from each other.

Supplementary Table 2. Liver total fatty acids profile in LBW swine fed experimental diets at 12 weeks of age

	LBW-CHF	LBW-LFDairy	LBW-HFDairy	*P*
Total SFA	45.60±0.61	45.86±0.41	47.02±0.38	0.565
Total MUFA	17.35±0.60	17.30±0.93	16.63±0.70	0.795
Total PUFA	36.47±0.40	36.23±0.61	35.72±0.55	0.907
C14:0	0.31±0.02[b]	0.37±0.03[b]	0.56±0.04[a]	<0.001
C15:0	0.24±0.02[b]	0.25±0.01[b]	0.32±0.01[a]	<0.001
C16:0	15.12±0.43	15.63±0.46	16.57±0.68	0.179
C16:1	0.66±0.05	0.69±0.06	0.63±0.04	0.744
C17:0	0.58±0.03	0.61±0.03	0.62±0.05	0.756
C18:0	29.23±0.70	28.95±0.79	28.71±0.90	0.902
C18:1	16.29±0.56	16.21±0.88	15.67±0.66	0.801
C18:2n6	15.41±0.47	15.30±0.36	14.88±0.27	0.581
C18:3n6	0.17±0.03	0.15±0.01	0.17±0.02	0.701
C18:3n3	0.19±0.01	0.17±0.01	0.17±0.01	0.249
C20:0	0.46±0.06	0.41±0.03	0.41±0.03	0.581
C20:1	0.23±0.01[a,b]	0.23±0.01[a]	0.19±0.01[b]	0.018
C20:2n6	0.48±0.01[a]	0.49±0.01[a]	0.41±0.02[b]	<0.001
C20:3n6	1.57±0.11	1.86±0.09	1.52±0.12	0.072
C20:4n6	10.08±0.29	9.98±0.48	10.69±0.35	0.373
C20:5n3	2.25±0.12[a]	1.83±0.10[a,b]	1.51±0.22[b]	0.011
C24:0	0.24±0.01	0.24±0.02	0.46±0.20	0.336
C24:1	0.17±0.01	0.17±0.01	0.14±0.01	0.116
C22:5n3	1.28±0.07	1.31±0.10	1.37±0.07	0.711
C22:6n3	5.05±0.34	5.15±0.43	5.01±0.37	0.965

CHF, control high-fat diet; HFDairy, high-fat dairy diet; LBW, low birthweight; LFDairy, low-fat dairy diet; MUFA, monounsaturated fatty acid; NBW, normal birthweight; PUFA, polyunsaturated fatty acid; SFA, saturated fatty acid. All values are expressed as means±SEM. A p value was considered as statistically significant when <0.05. Means sharing the same letter were not significantly different from each other.

Supplementary Table 3. Fasting plasma inflammatory markers in LBW and NBW swine fed different experimental diets

	NBW-Chow	NBW-CHF	LBW-CHF	LBW-HFDairy	LBW-LFDairy	*P*
IL-1α, pg/ml	9.7±2.6[a,b]	10.1±1.4[b]	16.3±7.3[a,b]	36.1±7.9[a]	16.6±4.8[a,b]	0.029
IL-1β, pg/ml	80.8±15.0[a,b]	74.7±9.7[b]	186.7±62.4[a,b]	310.4±77.2[a]	128.4±35.5[a,b]	0.031
IL-1Ra, pg/ml	207.5±56.3	200.6±25.6	307.6±34.8	378.5±47.9	314.0±48.9	0.051
IL-2, pg/ml	56.4±12.9	61.9±14.2	122.7±53.9	224.7±57.4	107.4±36.6	0.087
IL-4, pg/ml	140.2±48.9[b]	175.3±23.8[b]	312.3±169.1[a,b]	914.9±227.3[a]	456.4±137.0[a,b]	0.013
IL-6, pg/ml	34.9±7.2	31.0±4.9	60.7±26.5	103.8±27.7	46.7±13.7	0.135
IL-8, pg/ml	52.8±2.8	55.0±3.1	61.2±6.9	58.4±3.6	55.1±1.1	0.682
IL-10, pg/ml	160.9±35.4	149.7±39.1	356.8±155.7	604.5±173.7	392.9±124.2	0.157
IL-12, pg/ml	575.7±156.8	669.3±72.8	757.3±134.9	666.8±87.9	519.3±61.7	0.548
IL-18, pg/ml	628.0±73.0	582.3±94.2	1039±295.8	1472±310.7	866.7±220.2	0.110
TNF-α, pg/ml	59.7±12.5	76.6±6.6	72.7±8.9	90.6±16.2	80.6±10.7	0.518
IFN-γ, pg/ml	4290±1022	3893±429	3469±375.9	3421±368.7	2920±93.5	0.423
IL4/IFN-γ ratio	0.03±0.01[b]	0.05±0.01[b]	0.08±0.04[b]	0.30±0.06[a]	0.14±0.05[a,b]	0.001

CHF, control high-fat diet; HFDairy, high-fat dairy diet; IFN-γ, interferon-gamma; IL, interleukin; LBW, low birthweight; LFDairy, low-fat dairy diet; NBW, normal birthweight; TNF-α, tumor necrosis factor-alpha. All values are expressed as means ± SEM. A p value was considered as statistically significant when <0.05. Means sharing the same letter were not significantly different from each other.
